# Predictive Control of the Mobile Robot under the Deep Long-Short Term Memory Neural Network Model

**DOI:** 10.1155/2022/1835798

**Published:** 2022-09-21

**Authors:** Lan Zheng

**Affiliations:** The School of Civil Engineering, Harbin University, Harbin 150086, China

## Abstract

At present, there is a phenomenon of network data packet loss in the trajectory tracking control system, which will degrade or even destabilize the system's performance. Therefore, this work first explains the theory of the deep long-short term memory (LSTM) neural network model, the kinematic model of mobile robots, and the trajectory tracking error model. The reasons for data packet loss in the control system are analyzed. Second, a prediction model based on the LSTM network is designed according to the theory mentioned above. Finally, the training effect of the LSTM model and the robot trajectory tracking effect based on the model are tested by setting up simulation experiments. The research results are as follows: (1) The pose test error of the mobile robot will eventually tend to zero through the simulation curve generated by the pose parameters (*x*, *y*, *θ*) of the mobile robot. (2) The trajectory tracking error of the deep LSTM neural network prediction and compensation method with the packet loss rate of 5% is less than that with the packet loss rate of 10%. (3) The linear velocity *υ* of the mobile robot based on the prediction model of the LSTM network varies greatly but is always in the interval (−2, 2). Its angular velocity *ω* initially fluctuates greatly but gradually tends to zero after about 13 s. (4) When the prediction model tracks the trajectory of the robot, the horizontal position *x*, the vertical position *y*, and the angle *θ* coincide with the reference trajectory. The exploration is conducted to provide a reference for the research on data packet loss in the networked mobile robot trajectory tracking system.

## 1. Introduction

### 1.1. Research Background and Motivations

Mobile robots can be divided into wheeled, bipedal, crawler, and crawling types according to different moving methods, and wheeled mobile robots are the most widely used [1]. The wheeled mobile robot is a typical nonholonomic mechanical system with nonlinear, underactuated, and drift-free characteristics, which brings great challenges to the research on its motion control. In particular, the trajectory tracking problem of mobile robots has become one of the key technologies [2]. Its trajectory tracking error system is usually a nonlinear strong coupling system, which does not meet the necessary conditions of Brockett. Mobile robots inevitably come into contact with the external environment during the movement process, and there are problems such as model uncertainty and external disturbances [3]. In addition, some scholars have proposed solutions to the problem of data packet loss in the networked mobile robot trajectory tracking system. For example, the virtual polling algorithm implemented at the application layer improves the network performance by reducing the degree of data conflict. However, this method does not consider the situation that the wireless channel will produce packet loss. Using the method of setting the transmission protocol to reduce the error caused by the unreliable wireless channel effectively suppresses the influence of the wireless channel, but does not compensate for the data packet loss caused by medium access [4]. Besides, there is a method to modify the controller output to improve the control performance by setting a gain schedule according to the current network conditions. Although it considers the influence of the wireless channel and medium access control, its statistical estimation algorithm for network data packet loss needs to be improved [5]. In general, most researchers are committed to optimizing existing network communication protocols, reducing the occurrence of network data packet loss and developing new network protocols to solve the impact of data packet loss on the performance of networked mobile robots [6]. Although these two methods can improve the system performance, the changes or innovations for network protocols have an enormous workload and high work difficulty. It is effective to analyze the impact of data packet loss in the existing network communication on the performance of the control system, improve and optimize the controller and propose a good algorithm to predict and compensate for data packet loss [7].

### 1.2. Research Objectives

**Figure 1:**
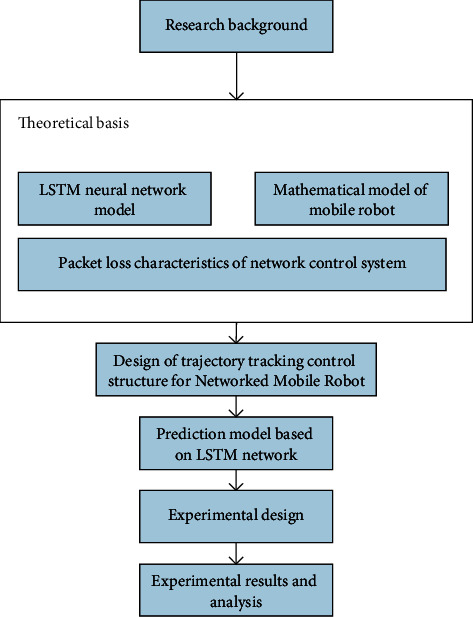
Structure of the work.

According to the content, the specific framework is given as shown in Figure 1.

This paper aims to use the LSTM neural network model to achieve high-precision prediction and control of mobile robot trajectories, thereby reducing some of the problems existing in mobile robots. This paper provides a reference value for further research on motion trajectory control.

## 2. Literature Review

The research on mobile robots began in the late 1960s, and Tianfu Innovation Institute was the first to begin research on mobile robots. At present, mobile robots have developed into an important branch of robotics [8]. In the aerospace field, there are lunar rovers and Mars rovers. The emergence of various reconnaissance robots and patrol cars in the military field, sweeping robots in the field of daily life, and hotel food delivery robots fully demonstrate the broad development prospects and application value of mobile robots [9]. The working conditions of the mobile robot have strong uncertainty according to the working field compared with the robot fixed in a position. Meanwhile, the mobile robot has the characteristics of unstructured, so its performance requirements are high. It needs to have the walking function, perception of the outside world, and some specific functions set by humans [10]. Researchers should conduct in-depth research on environmental perception, dynamic programming, and sensory information fusion [11].

Regarding the trajectory prediction research of mobile robots, Islam et al. combined differential flatness characteristics and integral sliding mode control and considered the dynamic characteristics of wheel actuators, which could realize trajectory tracking control of single-wheel mobile robots [12]. Barzegar-Kalashani et al. used a complete sliding mode controller for the first time in the research on trajectory tracking control of a two-wheeled mobile robot, which could effectively reduce the influence of uncertain factors and achieve a good tracking effect [13]. Yang et al. combined a neural network with a sliding mode control algorithm to ensure the stability of neural network adaptation, and they obtained appropriate equivalent control when the parameters of the robot model were unknown. This method could ensure that the output tracking error converged to zero [14]. Liang et al. designed an adaptive trajectory tracking control algorithm based on the kinematic error model of mobile robots according to the shortcomings of traditional trajectory tracking control laws. They verified that the algorithm could track the reference trajectory at a fast speed and has an excellent tracking control effect through experiments [15].

It is found that although the research on the trajectory tracking control of networked mobile robots with data packet loss has been developed, there are still many deficiencies by sorting out the literature. On the one hand, in the process of trajectory tracking control of mobile robots, the two sub-problems of motion planning and tracking control are usually solved separately. This results in that the external constraints are not considered when the reference trajectory of the mobile robot is given. The robot cannot reach the given input at some sampling moments, so it cannot achieve effective trajectory tracking. On the other hand, most of the current methods for improving the tracking performance of networked mobile robots are the development or optimization of network communication protocols with a heavy workload and high difficulty. It is urgent to propose an effective packet loss prediction compensation algorithm. The innovation lies in proposing a new prediction model and setting simulation experiments based on the original research on robot trajectory control. The new model is based on a long short-term memory (LSTM) neural network, which is optimized and integrated into robot trajectory prediction control. This work has a reference value in the research of robot trajectory prediction control.

## 3. Research Methodology

### 3.1. LSTM Neural Network Model

#### 3.1.1. The Concept of the LSTM Model

**Figure 2:**
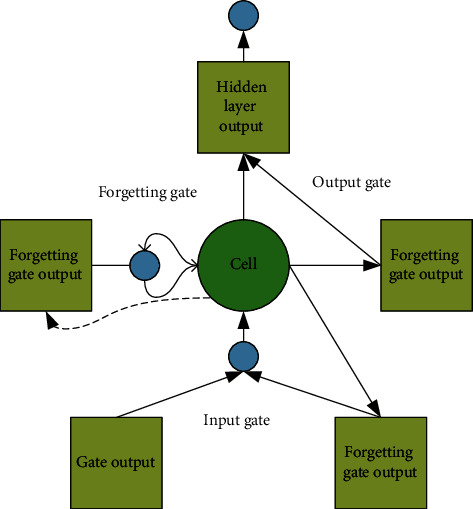
Schematic diagram of the LSTM unit structure.

With the continuous development of deep learning, the types of network architectures are also increasing, which are mainly divided into two categories: deep discriminative models and deep generative models [16]. This work uses deep learning for network data packet loss prediction compensation, so the deep LSTM neural network in the deep discriminant model is adopted. The LSTM unit is a variant of the most widely used recurrent neural network (RNN). It inherits the characteristics of most of the RNN models and solves the problem that RNN is difficult to train due to long-term dependencies [17]. LSTM units realistically represent or simulate the cognitive processes of human behavior, logical development, and neural organization. It is suitable for handling problems that are highly related to time series, such as machine translation, dialogue generation, encoding, and decoding [18]. The LSTM unit structure is shown in Figure 2.

Figure 2 shows the overall research framework. For LSTM, the model cell adds a cell state to store the long-term state, so it can effectively solve the long-term dependency problem. The key to the LSTM unit is how to effectively control the cell state. Three gates are added: input gate, forget gate, and output gate. The essence of the gate is a fully connected layer, the input is a vector, and the output is a real vector between zero and one. The elements in the gate's output vector are in turn multiplied by the vector to be controlled. When the output of the gate equals zero, its product with any vector is zero. At this point, no information can pass through. When the output of the gate is equal to one, its product with any vector is unchanged. At this point, any information can pass [19]. LSTM cells control the cell state through input gates and forget gates. The input gate determines how much of the current input is stored in the cell. The forget gate determines how much of the cell state at the previous moment is retained in the current cell state. The output gate determines how many cell states are passed to the current output of the network [20].

#### 3.1.2. Features of the LSTM Model

In the process of training the network model using the gradient descent method, the traditional RNN's weight update strategy is the correct direction based on the weight at the end of the output sequence. The weight changes only depend on the sequence input at the most recent moments, and the input data from a long time ago has little effect. Besides, the network has poor long-term memory function, and the training results are inclined to new input information [21].

During the training process of the LSTM model, the stochastic gradient descent method is used to optimize the network parameters. This is the standard optimization method recently adopted to train deep neural networks, and it is easy to implement [22]. At each iteration, the objective function is run on a subset of the training set, which is called the mini-batch. The gradient of the mini-batch objective function is obtained by back-propagating the neural network parameters. The step size is determined by the average value of the past step size and the current gradient, and the weight of the past step size is determined by the momentum hyperparameter. The parameters (weights and biases) are updated according to the new step size and scaled by the learning rate. The new mini-batch is randomly determined from the training set and used for the next iteration. The learning rate is decreased during optimization [23].

### 3.2. Mathematical Model of the Mobile Robot

#### 3.2.1. Kinematics Model of the Mobile Robot

**Figure 3:**
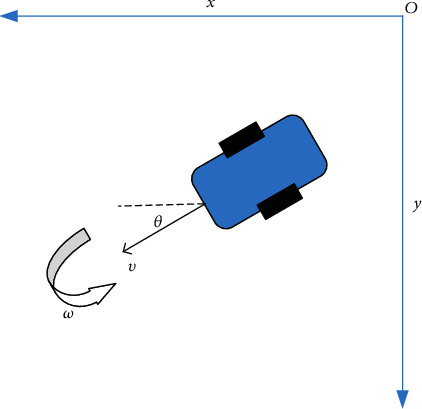
Schematic diagram of a two-wheel differential mobile robot.

The kinematic model of a mobile robot directly reflects the relationship between its pose state and control input, and it is the most intuitive mathematical model [24]. The research object is a typical two-wheel differential mobile robot. This kind of mobile robot is composed of a balance wheel and two driving wheels, and the mobile robot realizes the rotation of the car body through the differential speed of the two driving wheels. The balance wheel only has a supporting function and cannot provide power for the mobile robot. Its specific structure is displayed in Figure 3.

According to the analysis of the collected literature, the balance wheel does not provide power, so the balance wheel can be ignored when the kinematic model equation of the mobile robot is derived. Besides, its number will not affect the kinematic equation form. The kinematic model of the two-wheel differential mobile robot can be expressed as

(1) $$\begin{array}{c} \left[\begin{array}{c} \dot{x}\\ \dot{y}\\ \dot{\theta } \end{array}\right]=\left[\begin{array}{cc} \cos\;\theta & 0\\ \sin\;\theta & 0\\ 0 & 1 \end{array}\right]\left[\begin{array}{c} \upsilon \\ \omega \end{array}\right]\text{,} \end{array}$$

(*x*, *y*) is the center position coordinate of the mobile robot. *θ* is the direction angle, and it is also the angle between the forward direction and the *x*-axis. *υ* and *ω* are control input signals in the kinematic model, and their physical meanings are the linear and angular velocities of the mobile robot [25].

#### 3.2.2. Trajectory Tracking Error Model of the Mobile Robot

**Figure 4:**
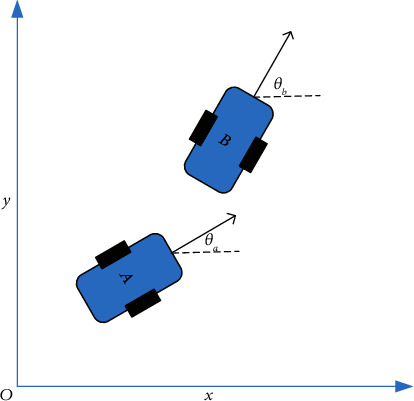
Schematic diagram of pose coordinate error.

The mobile robot task trajectory tracking error model established here is demonstrated in Figure 4.

In Figure 4, point *A* (*x*_*a*_, *y*_*a*_) is the center position of the target mobile robot. *θ*_*a*_ is the direction angle. They together constitute the pose state vector of the target mobile robot [*x*_*a*_, *y*_*a*_, *θ*_*a*_]^*T*^. Point *B* (*x*_*b*_, *y*_*b*_) is the center position of the reference mobile robot. *θ*_*b*_ is the direction angle. The pose state vector is [*x*_*b*_, *y*_*b*_, *θ*_*b*_]^*T*^. At this time, the pose error between the target robot and the reference robot can be expressed as

(2) $$\begin{array}{c} \left[\begin{array}{c} x_{e}\\ y_{e}\\ \theta_{e} \end{array}\right]=\left[\begin{array}{ccc} \cos\;\theta & \sin\;\theta & 0\\ -\sin\;\theta & \cos\;\theta & 0\\ 0 & 0 & 1 \end{array}\right]\left[\begin{array}{c} x_{b}-x_{a}\\ y_{b}-y_{a}\\ \theta_{b}-\theta_{a} \end{array}\right]\text{.} \end{array}$$

Then, equation (3) can be acquired as

(3) $$\begin{array}{c} \left\{\begin{array}{c} x_{e}=\left(x_{b}-x_{a}\right)\cos\;\theta +\left(y_{b}-y_{a}\right)\sin\theta \text{,}\\ y_{e}=-\left(x_{b}-x_{a}\right)\sin\;\theta +\left(y_{b}-y_{a}\right)\cos\theta \text{,}\\ \theta_{e}=\left(\theta_{b}-\theta_{a}\right)\text{.} \end{array}\right. \end{array}$$

The trajectory tracking error model of the mobile robot can be obtained by derivation of (3).

(4) $$\begin{array}{c} \left[\begin{array}{c} \dot{x_{e}}\\ \dot{y_{e}}\\ \dot{\theta_{e}} \end{array}\right]=\left[\begin{array}{c} \upsilon_{b}\cos\theta_{e}-\upsilon +y_{e}\omega \\ \upsilon_{b}\sin\theta_{e}-x_{e}\omega \\ \omega_{b}-\omega \end{array}\right]\text{.} \end{array}$$

The trajectory tracking control problem of the mobile robot can be transformed into the stabilization problem of the trajectory tracking error model shown in (4) based on the new pose error state vector [*x*_*e*_, *y*_*e*_, *θ*_*e*_]^*T*^. All the items in the trajectory tracking error vector [*x*_*e*_, *y*_*e*_, *θ*_*e*_]^*T*^ tend to be zero by designing appropriate *υ* and *ω* control laws. When the system reaches [*x*_*e*_, *y*_*e*_, *θ*_*e*_]=[0,0,0], *x*_*b*_=*x*_*a*_, *y*_*b*_=*y*_*a*_, and *θ*_*b*_=*θ*_*a*_, the mobile robot trajectory tracking control task is completed [26].

### 3.3. Packet Loss Characteristics of the Network Control System

#### 3.3.1. Reasons for packet loss

In a network control system, each network node is frequently exchanging information. During network transmission, data collision or competition between nodes may occur, which will result in data loss during transmission. The network protocol has a retransmission mechanism to solve the problem of packet loss, but packet retransmission timeout may also occur when the system load is too heavy and there are many nodes in the network for data exchange. Packet loss can still happen. However, the phenomenon of packet loss sometimes occurs in the practical application of network control systems, which will reduce the system performance. Different network control systems will set a range of packet loss thresholds. Once the packet loss rate exceeds the threshold, the system will oscillate erratically, resulting in packet loss during network transmission [27]. There are three main reasons for packet loss.

A network node in the system has a communication failure. When the processor of a node in the network fails, the buffer where the data packet is located will be emptied, resulting in packet loss.

The packet transmission task in the system is too frequent. When the system communicates frequently, data conflicts inevitably occur. At this time, each node in the network competes for the right to use the network bandwidth, and the loss of data packets will occur. Although there is a retransmission mechanism, the destination node usually directly discards the packets that have not been transmitted beyond the retransmission time threshold.

There is channel interference in the network. The external environmental factors that the actual system is exposed to affect the transmission quality of the data packets in the channel. Channel interference in the network may cause disorder or loss of physical signals, resulting in distortion of data packets after reaching the destination node. At this time, valid data cannot be recovered through corresponding algorithms, and data packets are lost [28].

#### 3.3.2. Description and Analysis of Network Packet Loss Characteristics

At present, the following three methods are mainly used to describe the data packet loss characteristics of the network control system. The first is statistical methods. For example, the probability distribution and the packet loss rate of data packets are assumed by using random system theory and switching system theory. The second is the theory of variable-delay systems. The total number of data packet losses between two sampling times needs to be given. The third is the theory of switched systems or predictive control theory. The sampling moment of packet loss in the system is regarded as the disconnection of the network transmission channel. A switching system with dynamic switches is used to represent the network control system structure of packet loss [29].

**Figure 5:**
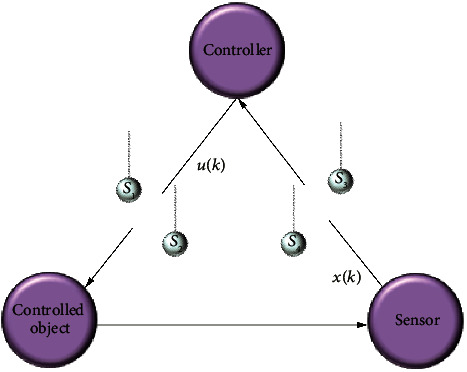
Simplified structure of the network control system.

In the network control system, serial communication is used for information transmission among the controller, the controlled object, and the sensor. Each node device in the system shares the network channel bandwidth. In the case of frequent system communication, errors occur in the process of data packet transmission or reception due to the competition of each node for the right to use the network bandwidth. When the data packet does not reach the receiver within the specified time, it is called packet loss [30]. When data loss occurs in the system, the system information transmission channel is temporarily disconnected. Valid information at some sampling moments is not transmitted to the receiver in time. This will directly affect the structure and parameters of the system, causing system performance degradation. For the problem of data packet loss, although most network control systems have robustness, it is only limited to the number of data packet losses within the allowable threshold range. If this threshold is exceeded, it will seriously affect the control performance of the system and even make the system unstable [31]. Therefore, Figure 5 shows the structure obtained by simplifying the network control system with packet loss.

The switch can be opened and closed, and its state indicates whether there is a packet loss. When the switch is closed (*S*_*2*_, *S*_*4*_), the data packet at the sampling moment can be transmitted smoothly. When the switch is off (*S*_*1*_, *S*_*3*_), the data packet is lost at the sampling moment. At this time, the transmission size at the previous moment is regarded as the packet loss data at this moment or the data at this moment is directly set to zero, which are called the keep-input strategy and the zero-input strategy, respectively. The keep-input strategy is widely used, and its mathematical expression is as follows.

When the switch is located at *S*_*1*_ and *S*_*3*_, there i

(5) $$\begin{array}{c} \left\{\begin{array}{c} \bar{u}\left(k\right)=u\left(k-1\right)\text{,}\\ \bar{x}\left(k\right)=x\left(k-1\right)\text{.} \end{array}\right. \end{array}$$

When the switch is located at *S*_*1*_ and *S*_*4*_, there is

(6) $$\begin{array}{c} \left\{\begin{array}{c} \bar{u}\left(k\right)=u\left(k-1\right)\text{,}\\ \bar{x}\left(k\right)=x\left(k\right)\text{.} \end{array}\right. \end{array}$$

When the switch is located at *S*_*2*_ and *S*_*3*_, there is

(7) $$\begin{array}{c} \left\{\begin{array}{c} \bar{u}\left(k\right)=u\left(k\right)\text{,}\\ \bar{x}\left(k\right)=x\left(k-1\right)\text{.} \end{array}\right. \end{array}$$

When the switch is located at *S*_*2*_ and *S*_*4*_, there is

(8) $$\begin{array}{c} \left\{\begin{array}{c} \bar{u}\left(k\right)=u\left(k\right)\text{,}\\ \bar{x}\left(k\right)=x\left(k\right)\text{,} \end{array}\right. \end{array}$$

$\bar{x}\left(k\right)$ represents the input value, and $\bar{u}\left(k\right)$ represents the output value. Besides, a new variable*z*(*k*) is defined as

(9) $$\begin{array}{c} z\left(k\right)=\left[\begin{array}{c} x\left(k\right)\\ \bar{x}\left(k\right)\\ \bar{u}\left(k\right) \end{array}\right]\text{.} \end{array}$$

The network control system model with data packet loss can be expressed as

(10) $$\begin{array}{c} z\left(k+1\right)=\Phi_{s}z\left(k\right)\text{.} \end{array}$$

However, only adopting the keep-input strategy and the zero-input strategy to compensate for the data packet loss of the network control system cannot solve the problem of system performance degradation or even system instability caused by data packet loss. Reasonable methods need to be proposed to compensate for the negative impact of packet loss. Some scholars have proposed the method of predictive control to realize the prediction of missing data, and they use the predicted value obtained by this method to replace the lost control input value. The main problem with the predictive control method is the need to define extended vectors to assist in the design of the closed-loop system controller. The introduction of the extended vector increases the conservatism of the entire closed-loop control system, so it remains to find a good predictive compensation method to overcome this adverse effect.

### 3.4. Trajectory Tracking Control of the Networked Mobile Robot

#### 3.4.1. Problem Description

**Figure 6:**
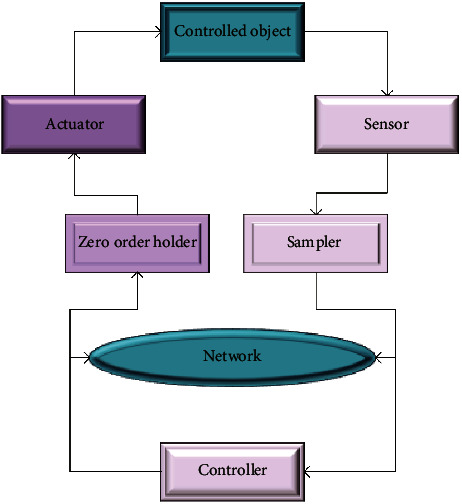
Network control system model with packet loss.

The model structure of the networked mobile robot control system with the data packet loss problem studied here is shown in Figure 6.

In this model, only the impact of packet loss on the system performance is considered during network transmission, and the sensor and controller are assumed to be time-driven and event-driven, respectively.

#### 3.4.2. System Model

Based on the above analysis of the system packet loss characteristics and causes, the following assumptions are made to ensure the enforceability of the packet loss prediction and compensation method proposed here.

In the control loop, the sensor sends data packets to the controller with a constant sampling period. In practical industrial design and implementation, a time-driven approach is usually used to ensure a constant sampling period [32–37].

The sensors, controllers, and controlled objects in the control loop are time-synchronized through time-stamping technology to deal with the problem of possible out-of-order data packets [38–42].

In each sampling period, the deep neural network makes predictions for a single data packet obtained by the sensor and sends this data packet to the controller [43–48].

**Figure 7:**
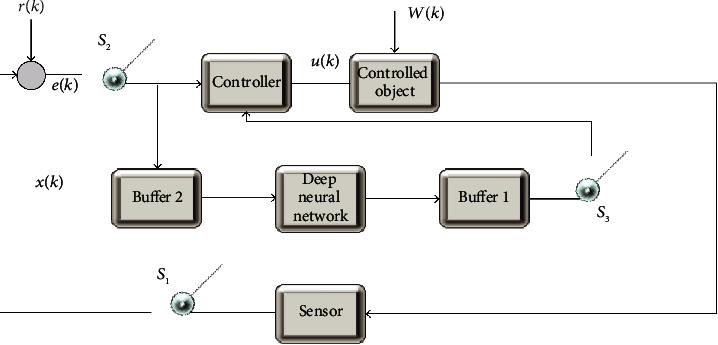
Structure of the mobile robot trajectory tracking control system based on the deep neural network.

Based on the above assumptions, the structure of the networked mobile robot trajectory tracking control system under the deep neural network model reported here is revealed in Figure 7.

In Figure 7, *r*(*k*) represents the reference pose of the mobile robot obtained by the partial point-taking method. *x*(*k*) represents the actual motion pose of the mobile robot measured by the sensor. *e*(*k*) represents the deviation between the actual pose and the reference pose of the mobile robot. This deviation serves as an input to the controller. *u*(*k*) is the control amount of the mobile robot, including the linear velocity *υ* and the angular velocity *ω*. *W*(*k*) is the interference noise of the external environment. If no data packet loss occurs at the current sampling time, *S*_*1*_ and *S*_*2*_ are closed, and *S*_*3*_ is disconnected. If data packet loss occurs at the current sampling time, *S*_*1*_ and *S*_*2*_ are disconnected, and *S*_*3*_ is closed [49–54].

### 3.5. Prediction Model Based on the LSTM Network

**Figure 8:**
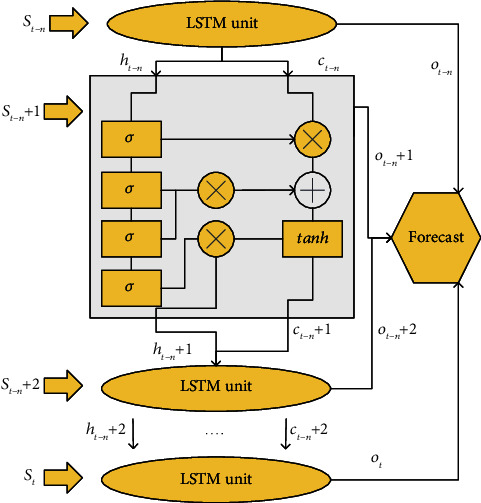
Mobile robot trajectory prediction model based on the LSTM network.

According to the above LSTM model, the mathematical model of the mobile robot, and the analysis of the data packet loss characteristics, the prediction model based on the LSTM network proposed here is shown in Figure 8.

The mathematical description of the LSTM unit is as follows:

(11) $$\begin{array}{c} f_{t}=\sigma \left(W_{f}x_{t}+U_{f}h_{t-1}+b_{f}\right)\text{,} \end{array}$$

(12) $$\begin{array}{c} i_{t}=\sigma \left(W_{i}x_{t}+U_{i}h_{t-1}+b_{i}\right)\text{,} \end{array}$$

(13) $$\begin{array}{c} o_{t}=\sigma \left(W_{o}x_{t}+U_{o}h_{t-1}+b_{o}\right)\text{,} \end{array}$$

(14) $$\begin{array}{c} c_{t}=f_{t}⊙c_{t-1}+i_{t}⊙\tan\;h\left(W_{c}x_{t}+U_{c}h_{t-1}+b_{c}\right)\text{,} \end{array}$$

(15) $$\begin{array}{c} h_{t}=o_{t}⊙\tan\;h\;\left(c_{t}\right)\text{.} \end{array}$$

In Equations (11)–(15), *x*_*t*_ is the input vector at time *t*, *f*_*t*_, *i*_*t*_, *o*_*t*_, *c*_*t*_, and *h*_*t*_ represent forget gate, input gate, output gate, cell state, and hidden layer output, respectively. *W*_*f*_, *W*_*i*_, *W*_*o*_, and *W*_*c*_ are input weights. *U*_*f*_, *U*_*i*_, *U*_*o*_, and *U*_*c*_ are cycle weights. *b*_*f*_, *b*_*i*_, *b*_*o*_, and *b*_*c*_ are biased values. *σ* and tanh are the *Sigmoid* and tanh activation functions, respectively. ⊙ represents the dot multiplication operation.

## 4. Experimental Design and Performance Evaluation

### 4.1. Datasets Collection

This work will use the deep LSTM neural network toolbox is used to calculate the unmodeled parameters of the model, and 1100 groups of data with two inputs and three outputs are randomly generated. The first 1000 groups are used to train the neural network, and the last 100 groups are used to test the modeling error. The whole dataset comes from the output of the deep LSTM neural network toolbox.

### 4.2. Experimental Environment

To verify the prediction model of robot trajectory tracking error based on the LSTM neural network without different packet loss rates, this paper builds and simulates the model by using the toolbox in MatLab application.

### 4.3. Parameters Setting

In the process of modeling, the training goal of the model is set to 0.001, and other parameters are the default values.

### 4.4. Performance Evaluation

#### 4.4.1. LSTM Network Model Training Effect

**Figure 9:**
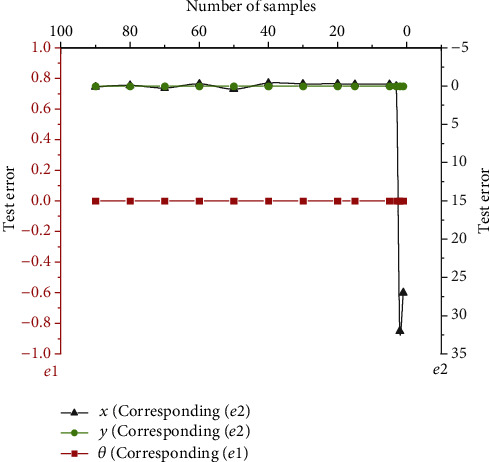
Prediction model training results based on the LSTM network.

The prediction model based on the LSTM network is trained by setting up simulation experiments.  Figure 9 reveals the results.

The meaning of the upper coordinate axis in Figure 9 refers to the serial number of the training samples collected in the experiment. In Figure 9, three lines with different colors represent the pose parameters (*x*, *y*, *θ*) of the mobile robot. The simulation curves generated by these three variables show that the pose test error of the mobile robot will eventually tend to zero. Therefore, it can be determined that the trained LSTM neural network can approximate the mobile robot model infinitely. The accuracy and efficiency are also high, which lays a solid foundation for the trajectory tracking controller to have a good tracking performance.

#### 4.4.2. Robot Trajectory Tracking Control Effect Based on the LSTM Model

**Figure 10:**
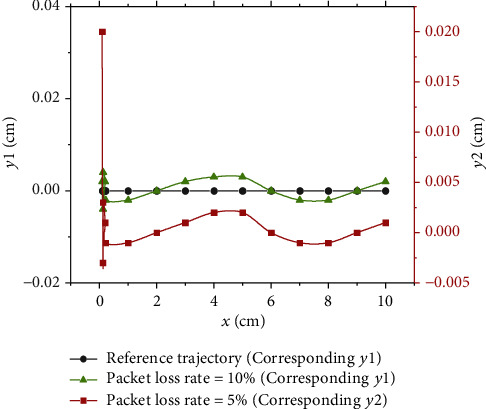
Trajectory tracking error results under different packet loss rates.

*(1) Robot Trajectory Tracking Error Under Different Packet Loss Rates.* The trajectory tracking error results of the prediction model based on the LSTM model under different packet loss rates are shown in Figure 10.

Figure 10 indicates that the trajectory tracking error of the deep LSTM neural network prediction compensation method when the packet loss rate is 5% is smaller than that of the deep LSTM neural network prediction compensation method when the packet loss rate is 10%. Generally, this method can complete the corresponding trajectory tracking task.

**Figure 11:**
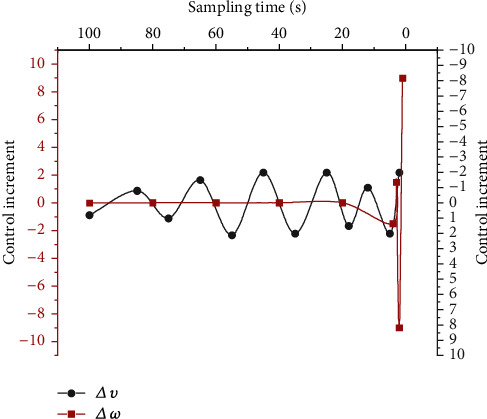
Control increment changes for mobile robots.

(2) Predictive Control Effect Of the Robot Based on the LSTM Network.  Figure 11 shows the result of the change of control amount when the robot based on the LSTM network moves through the simulation experiment.

Figure 11 reveals that the linear velocity *υ* of the mobile robot based on the prediction model of the LSTM network varies greatly but is always in the interval (−2, 2). The angular velocity *ω* initially fluctuates greatly but gradually tends to zero after about 13 s. In conclusion, the control increment changes of the mobile robot under this model are stable. The prediction effect of the model is further verified.

**Figure 12:**
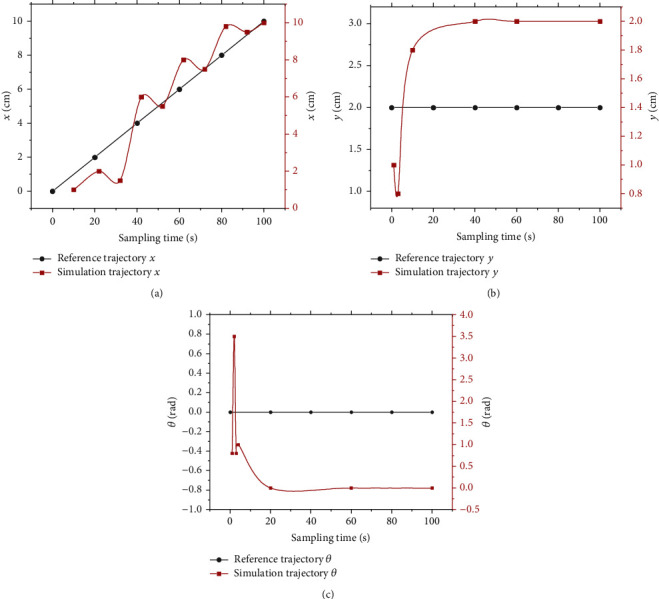
Mobile robot trajectory tracking status. (a) The lateral displacement tracking the state of the mobile robot; (b) the longitudinal displacement tracking the state of the mobile robot; (c) rotation angle tracking the state of the mobile robot.

*(3) Robot Linear Trajectory Tracking Effect Based on the LSTM Network.* Figure 12 shows the effect of robot linear trajectory tracking based on the LSTM network through simulation experiments.

Figure 12 shows that each variable of the predicted trajectory has good prediction accuracy when the prediction model is used to track the robot's moving trajectory. It suggests that the lateral position *x*, longitudinal position *y*, and angle *θ* predicted by the LSTM model basically coincide with the reference trajectory provided. The above analysis shows that the model has a good prediction effect on the trajectory of the robot, and the effect of the prediction model is finally verified.

## 5. Discussion

On the one hand, the results show that the LSTM model is different from other network models. The training time of the model is less because of the particularity of its structure, so it has higher accuracy, which provides a reliable basis for the high-precision prediction of the robot trajectory. In addition, the model can simulate and predict the motion trajectory of the robot with high precision by reducing the packet loss rate in the network control system. On the other hand, based on the previous research on mobile robot control, this work uses the deep learning algorithm to design its control model. The selected deep learning algorithm is the LSTM algorithm, which has a good performance for predictive control. Therefore, this algorithm is selected as the theoretical support to complete the prediction of mobile robot trajectory. The basis is the existing problem of trajectory predictive control of mobile robots.

## 6. Conclusion

### 6.1. Research Contribution

This paper presents a robot trajectory prediction model based on an LSTM neural network combined with the mathematical model of mobile robots and the phenomenon of data packet loss. The research object is the existing problems of trajectory prediction control of mobile robots. The author presents a robot trajectory prediction model based on the LSTM neural network combined with the mathematical model of mobile robots and the phenomenon of data packet loss. After the model is tested by setting up simulation experiments, the following conclusions are drawn. First, the trained LSTM neural network can approximate the mobile robot model infinitely, not only with high accuracy but also with high efficiency. Second, in the case of different packet loss rates, the model can still complete the corresponding curve tracking task well. Third, the control increment changes of the mobile robot under this prediction model are stable. Fourth, when the prediction model tracks the movement trajectory of the robot, the specific variables in it are coincident with the reference trajectory.

### 6.2. Future Works and Research Limitations

The deficiency lies in that, on the one hand, only the straight-line form is selected for the reference trajectory when testing the model, which does not reflect the model testing effect under other reference trajectory forms. The purpose is to design a trajectory prediction model by combining neural network technology and the kinematic model to improve the accuracy and quality of trajectory prediction. On the other hand, the effect of the LSTM prediction model proposed here has only been reflected in the simulation experiment, but it has not been applied to the mobile robot, and its effectiveness needs to be further studied. Subsequently, the reference trajectory in the form of the sine curve will be set to further verify the effect of the model. Additionally, it will be introduced into a specific mobile robot example to further verify the effect of the model.

## Data Availability

The data are available from the corresponding author upon request.
